# Facile Fabrication of Dumbbell-Like β-Bi_2_O_3_/Graphene Nanocomposites and Their Highly Efficient Photocatalytic Activity

**DOI:** 10.3390/ma11081359

**Published:** 2018-08-06

**Authors:** Jun Yang, Taiping Xie, Chenglun Liu, Longjun Xu

**Affiliations:** 1 Chongqing Key Laboratory of Environmental Materials & Remediation Technologies, Chongqing University of Arts and Sciences, Yongchuan 402160, China; bbyangjun@foxmail.com; 2 Chongqing Key Laboratory of Extraordinary Bond Engineering and Advanced Materials Technology (EBEAM), Yangtze Normal University, Chongqing 408100, China; 3 State Key Laboratory of Coal Mine Disaster Dynamics and Control, Chongqing University, Chongqing 400044, China; xlclj@cqu.edu.cn; 4 College of Chemistry and Chemical Engineering, Chongqing University, Chongqing 401331, China

**Keywords:** Dumbbell-like β-Bi_2_O_3_, photocatalysis, β-Bi_2_O_3_/GN, graphene-based composite

## Abstract

β-Bi_2_O_3_ decorated graphene nanosheets (β-Bi_2_O_3_/GN) were prepared by a facile solution mixing method. The crystal structure, surface morphology, and photo absorbance properties of the products were characterized by XRD, SEM, and UV-VIS diffuse reflection, respectively. Moreover, the effect of graphene content on photocatalytic activity was systematically investigated, and the results indicated that these composites possessed a high degradation rate of Rhodamine B (RhB), which was three times higher than that of bare β-Bi_2_O_3_ when graphene content was 1 wt %. This high photocatalytic activity was attributed predominantly to the presence of graphene, which served as an electron collector and transporter to efficiently lengthen the lifetime of the photogenerated charge carriers from β-Bi_2_O_3_.

## 1. Introduction

The semiconductor can be used as a photocatalyst due to its unique electronic component structure (a valence band full of electrons and an empty conduction band). Bi_2_O_3_ is a common important semiconductor material, which is widely used in various fields, such as electronic ceramics, sensors, and high-temperature superconductivity, to name a few [1,2]. As a photocatalyst, it has gained more and more attention [3]. Bi_2_O_3_ mainly has four crystal structures, including α, β, γ, and δ. Due to a lower band gap and a unique electronic structure, the β-Bi_2_O_3_ has higher photocatalytic activity than the other configurations of Bi_2_O_3_ [4,5]. However, the photocatalytic activity of pure Bi_2_O_3_ is still restricted. In order to further enhance its activity, researchers have made efforts, such as using Pt, Au, Ag, and other noble metals, to improve the conductivity of the electrons and reduce the recombination probability of charge carrier [6,7]. Chai et al. [8] used Bi_2_O_3_ as a precursor to prepare the BiOCl/Bi_2_O_3_ complex and finally form a heterojunction so as to significantly improve its activity in the photocatalytic degradation of pollutants.

In recent years, graphene-based semiconductor photocatalysts have gained great attention due to their highly efficient electronic conduction force, larger specific surface area, and good adsorption performance [9]. To date, various graphene–semiconductor composites with enhanced photocatalytic performance have been designed, such as graphene–TiO_2_ nanocomposite [10], graphene/zirconium oxide [11] and RGO–Bi_2_O_3_ nanocomposite [12]. As far as we know, only a few research studies with graphene-based Bi_2_O_3_ used as a photocatalyst have been reported. For example, Som et al. [13] and Maruthamani et al. [14] introduced a co-precipitation route to prepare GO/α-Bi_2_O_3_ or rGO/Bi_2_O_3_ rods. Cao et al. [15] explored an organic electrolyte-assisted method to prepare the GR/β-Bi_2_O_3_ composites. Therefore, in this paper, we consider it important to undertake the green chemical synthesis of the composites; a simple method of solution mixing and thermal reduction was used to prepare β-Bi_2_O_3_ decorated graphene nanosheets (β-Bi_2_O_3_/GN) in one step. Rhodamine B (RhB) is used as the model organic dye to investigate the activity of as prepared samples, as it is an important factor in environmental pollution and its degradation mechanism has been studied quite well [16,17]. In addition, the effect of the content of graphene was also studied systematically, and finally, a possible photocatalytic mechanism of β-Bi_2_O_3_/GN composite was proposed.

## 2. Experimental

### 2.1. Synthesis of Bi_2_O_3_/GN

Graphite oxide was prepared by a modified Hummers method [18]; then, the aqueous solution of graphene oxide (GO) could be obtained by ultrasonic stripping from graphite oxide for 1 h. β-Bi_2_O_3_-decorated graphene nanosheets (β-Bi_2_O_3_/GN) were prepared by a facile solution mixing method and thermal reduction. In a typical process, 8 mmol of Bi(NO_3_)_3_·5H_2_O was dissolved in 20 cm^3^ of nitric acid (1 mol/dm^3^); then, a different volume of GO (2 mg/dm^3^) was added dropwise into the solution, which was then continuously stirred for 30 min. Then, the above solution was added dropwise into 80 cm^3^ (0.6 mol/dm^3^) of saturated sodium carbonate solution. The reaction mixture was stirred for 5 h before filtration. After being washed by water and ethanol several times respectively, the whole product was dried at 60 °C for 10 h. The dried product was then transferred into the muffle furnace after being ground. Finally, the dried product was roasted at 360 °C for 10 min under the nitrogen atmosphere protection, then, it was natural cooled to room temperature to obtain the resultant product. Here, it was worth noting that the heating rate was 4 °C/min. During the reaction, GO was reduced to GN. Pure β-Bi_2_O_3_ was synthesized by the same experimental process, except that GO was not added.

### 2.2. Materials’ Characterization

The samples’ crystal structure was characterized by XRD (Bruker Advance D8, Cu Kα irradiation, Bruker, Germany). Scanning electron microscopy (SEM, JSM-7800F, Japan electronics, Japan) was used to observe the morphology of the prepared samples. The Fourier transform infrared spectroscopy (FTIR) spectra of samples were recorded on a 5DX FTIR (5DX, Nicolet. Co., Rhinelander, WI, USA) spectrometer using KBr powder-pressed pellets. The Brunauer–Emmett–Teller (BET) special surface area was determined through N_2_ adsorption at 77 K using an adsorption instrument (ASAP-2020, Micromeritics, Norcross, GA, USA). The UV-VIS diffuse reflectance spectra (UV-vis DRS) of samples were measured using a UV-VIS spectrophotometer (TU1901, Beijing Purkinje, Beijing, China).

### 2.3. Test of Photocatalytic Activity

The photodegradation test was carried out by using a 300-W xenon lamp (the corresponding emission spectrum see Figure S1) (CEL-HXF300, AULTT, Beijing, Country), the self-made circulating water system maintained the temperature of the reaction system at 25 ± 5 °C. RhB solution (50 cm^3^, 10 mg/dm^3^) containing 50 mg of catalyst was put in a glass beaker and stirred in the dark overnight to ensure adsorption–desorption equilibrium. After light illumination at regular time intervals, the absorbance of the RhB solution was monitored by a UV-VIS spectrophotometer.

## 3. Results and Discussion

### 3.1. Crystal Structure Characterization

**Figure 1:**
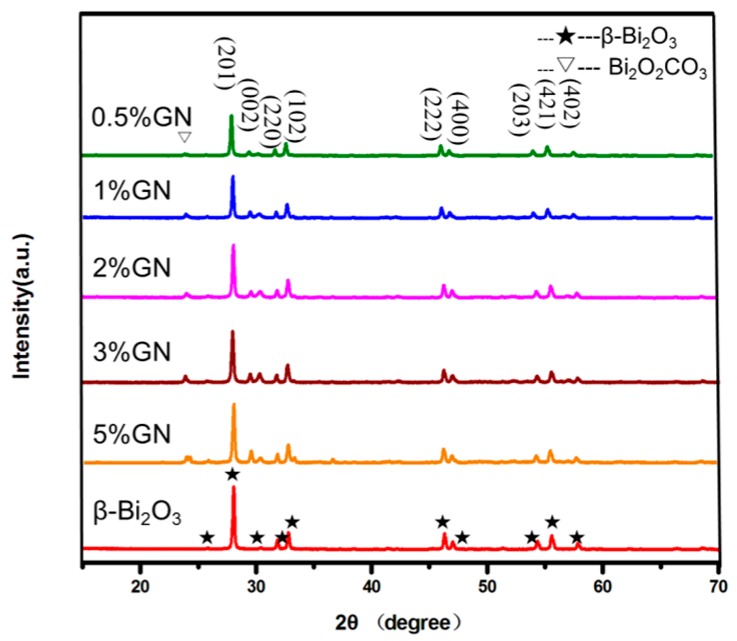
Diffraction patterns of pure β-Bi_2_O_3_ and hybrid composites with different mass ratios of graphene nanosheets (GN).

Figure 1 shows the XRD patterns of pure β-Bi_2_O_3_ and composites with different masses of GN. The peak positions of 27.9°, 31.7°, 32.6°, and 33.8° correspond to the crystal plane diffractions of (201), (002), (220), and (102) of the tetragonal β-Bi_2_O_3_ (JCPDS 27-0050), respectively. The diffraction peaks of the β-Bi_2_O_3_/GN sample and β-Bi_2_O_3_ are essentially the same, except for a few diffraction peaks of Bi_2_O_2_CO_3_ (the inverted triangle as shown in Figure 1. This may be due to the coating effect of GN, which caused Bi_2_O_2_CO_3_ to not be completely converted into β-Bi_2_O_3_ in the thermal decomposition process. However, Bi_2_O_2_CO_3_ has a similar electronic structure to Bi_2_O_3_, which is also a well-known photocatalyst. When Bi_2_O_2_CO_3_ was used as the photocatalytic material, it can play a synergistic effect. For example, Chai et al. reported that one β-Bi_2_O_3_/Bi_2_O_2_CO_3_ nanosheet composite exhibits much higher photodegradation activity than single phase [5]. It was worth noting that there were no significant carbon-related diffraction peaks in XRD, which is because of the low GN content and low diffraction intensity [19].

### 3.2. FTIR Spectra

**Figure 2:**
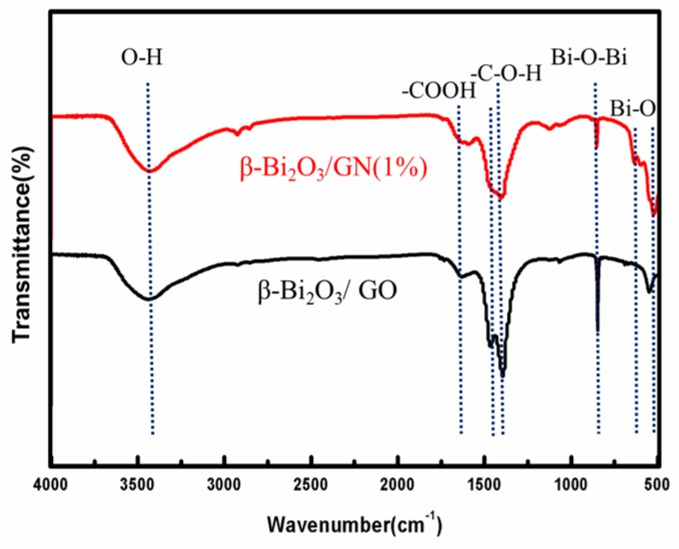
Fourier transform infrared (FTIR) spectra of β-Bi_2_O_3_/GN (1%) and β-Bi_2_O_3_/GO.

In order to better analyze the state of GN on the surface of β-Bi_2_O_3_, the FTIR of β-Bi_2_O_3_/GN (1%) and β-Bi_2_O_3_/GO are displayed in Figure 2. It can be seen that the composites both have a peak at 500–700 cm^−1^, which belongs to the telescopic vibration of the Bi–O bond of BiO_6_ octahedron, and the telescopic vibration peak at 840 cm^−1^ belongs to Bi–O–Bi [20]. In addition, the O–H stretching vibration of adsorbed water corresponds to 3450 cm^−1^, and the O–H stretching vibration peak in C–O–H corresponds to 1408 cm^−1^ [21,22]. The peaks at 1440–1630 cm^−1^ can be attributed to the antisymmetric stretching vibration and symmetry stretching vibration of the C=O bond in –COOH, respectively. It is worth noting that the C=O antisymmetric stretching vibration at 1450 cm^−1^ has disappeared after the thermal reduction, and what’s more, the peak intensity becomes weaker after reduction, indicating the effectiveness of the thermal reduction. The results of FTIR show the existence of GO and Bi_2_O_3_ in the composites, and GO is effectively reduced to GN after thermal reduction.

### 3.3. Surface Morphology Characterization

**Figure 3:**
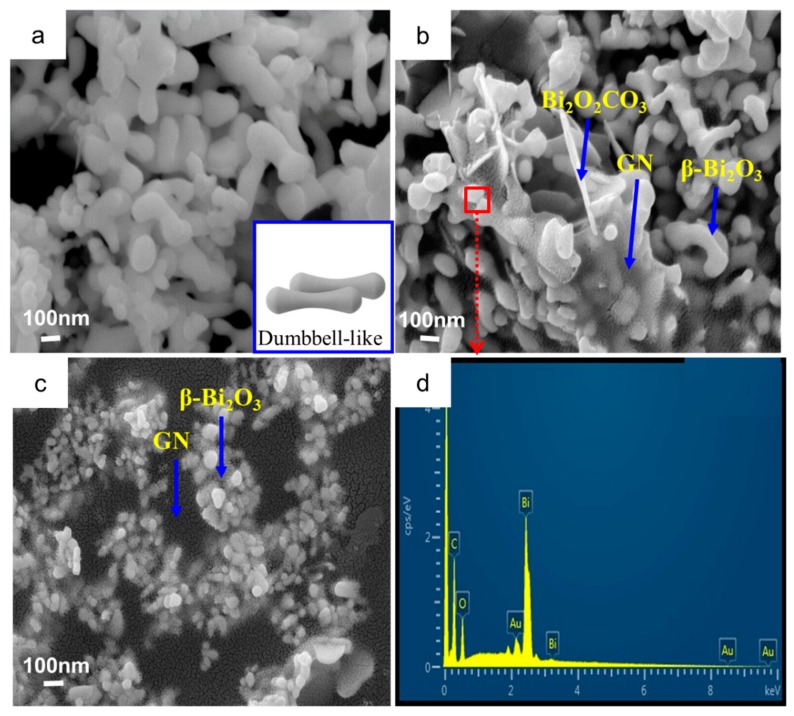
(**a**) SEM images of pure β-Bi_2_O_3_; (**b**,**c**) β-Bi_2_O_3_/GN(1%); and (**d**) EDS pattern of β-Bi_2_O_3_/GN.

Surface morphology of the samples was characterized by SEM (Figure 3). The pure β-Bi_2_O_3_ is as shown in Figure 3a. β-Bi_2_O_3_ has a dumbbell-like morphology (the insert is dumbbell). After the 1% GN was introduced, it can be clearly seen that the β-Bi_2_O_3_ was coated by GN (Figure 3b) or embedded in the GN sheets (Figure 3c). In Figure 3b, it can also be seen there is very little Bi_2_O_2_CO_3_, which was consistent with the XRD results. The elemental mapping (Figure 3d) of the ternary as-prepared β-Bi_2_O_3_/GN obtained by EDS (the mapped region highlighted with red frame in Figure 3b) indicates that the weight ratios of the elements are close to the Bi_2_O_3_ molar mass ratios. Meanwhile, only the peaks of C, O, Bi, Au were detected, which means that the as-prepared composites are composed of graphene and Bi_2_O_3._

### 3.4. Surface Areas and Pore Size Distributions

**Figure 4:**
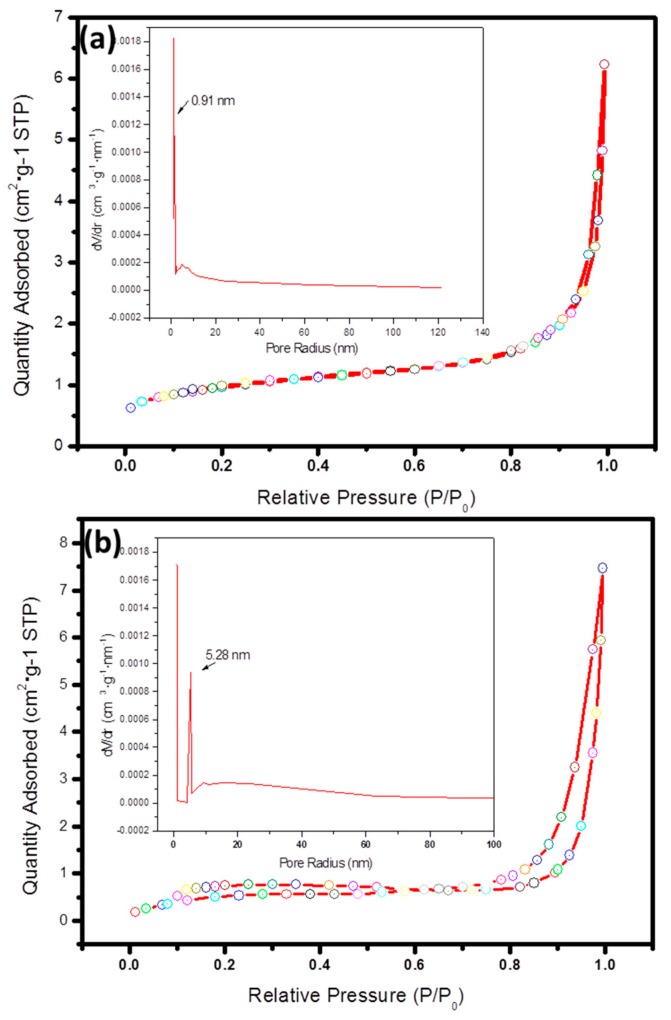
N_2_ adsorption–desorption isotherm of (**a**) β-Bi_2_O_3_ and (**b**) β-Bi_2_O_3_/GN (1%). Inset: the corresponding pore size distribution.

In general, the specific surface area of catalysts and its surface structure have significant influences on catalytic activity. Therefore, the specific surface analyzer is utilized to implement further research on the specific surface area and the pore diameter distribution of pure β-Bi_2_O_3_ and β-Bi_2_O_3_/GN (1%). Figure 4 shows the N_2_ adsorption–desorption isotherms and the corresponding curves of the pore size distribution (inset) for samples β-Bi_2_O_3_ and β-Bi_2_O_3_/GN (1%). According to the Brunauer–Deming–Deming–Teller (BDDT) classification, pure β-Bi_2_O_3_ isotherms can be categorized as type III (Figure 4a), which is convex to the P/P_0_ axis over its entire range, indicating that the pure β-Bi_2_O_3_ belong to a nonporous structure. Meanwhile, samples of β-Bi_2_O_3_/GN (1%) have isotherms of type IV, suggesting the presence of mesopores. Upon observing the pore diameter distribution diagram obtained from desorption isotherms, pore diameter mainly distributes in 5.28 nm (Figure 4b inset). The specific surface area of β-Bi_2_O_3_ and β-Bi_2_O_3_/GN (1%) are given by BET measurement as 3.37 m^2^/g, 4.53 m^2^/g, respectively, which conforms to the results. In other words, when graphene is introduced, mesopores begin to appear in samples and the specific surface area increases, which is because the prepared nanocomposite is composed of sheet-like graphene decorated with β-Bi_2_O_3_, similar to the previous report [23]. Consequently, the introduction of graphene can increase the specific surface area, and graphene may play a role in enhancing the photocatalytic activity.

### 3.5. UV-Vis Diffuse Reflectance and Photocatalytic Activity

**Figure 5:**
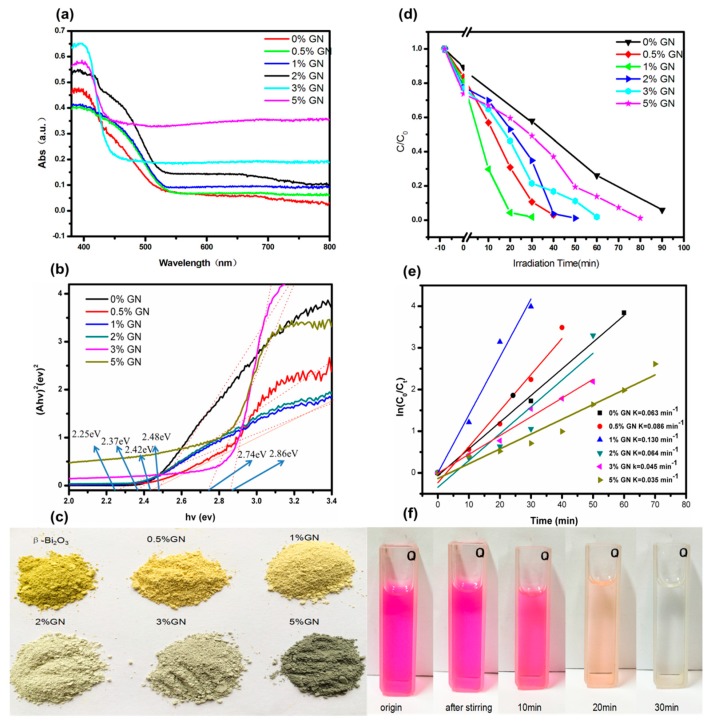
(**a**) The spectrogram of UV-VIS diffuse reflectance of GN with different mass ratios; and (**b**) corresponding band gap energy; (**c**) the chart of the corresponding sample color change; (**d**) the Rhodamine B (RhB) degradation rates of samples with different quantity ratios of GN; (**e**) kinetic curve of photodegradation; (**f**) color change chart of RhB solution by 1% GN degradation.

The photocatalytic activity tests are shown in Figure 5. From Figure 5a, the absorption of visible light by the samples can be seen as the content of GN increases, and the sample color gradually changing from yellow to deeper brown can also be seen in Figure 5c. This is another proof to the increasing absorption of visible light. The band gap of calculated β-Bi_2_O_3_ and composites are shown in Figure 5b. Although the 5% GN is not narrowest band gap, the absorbed threshold value of sample’s visible light region is significantly greater than the other samples. Therefore, it is hypothesized that the introduction of graphene can better absorb the visible light and enhance the photocatalytic activity.

The tests of photocatalytic degradation of RhB (Figure 5d) show that all of the composites have higher degradation rates than bare β-Bi_2_O_3_, suggesting that there is a synergistic effect between the GN sheet and β-Bi_2_O_3_ nanoparticles. The sequential dye degradation rates are as follows: 1% GN > 0.5% GN > 2% GN > 3% GN > 5% GN > bare β-Bi_2_O_3_. It seen that only the low content (1%) of GN is introduced, the dye degradation rate can be significantly increased by three times in comparison with the bare β-Bi_2_O_3_. The kinetics of photodegradation reaction are investigated in Figure 5e, and the results show that 1% GN composite has the highest constant photodegradation reaction rate, which is 0.130 min^−1^. With the increase of GN content, the dye degradation rate did not always increase; the activity of 5% GN was significantly less than that of 1% GN. The reason why the degradation ratio decreased as the content of graphene increased was that the introduction of a large mass of black graphene would result in a rapid decrease of the light absorption of the reaction solution [23,24]. Thus, in order to achieve an optimal photocatalytic performance, it is crucial to control the composition ratio in the nanocomposite of β-Bi_2_O_3_/GN.

### 3.6. Possible Mechanisms Speculation

**Figure 6:**
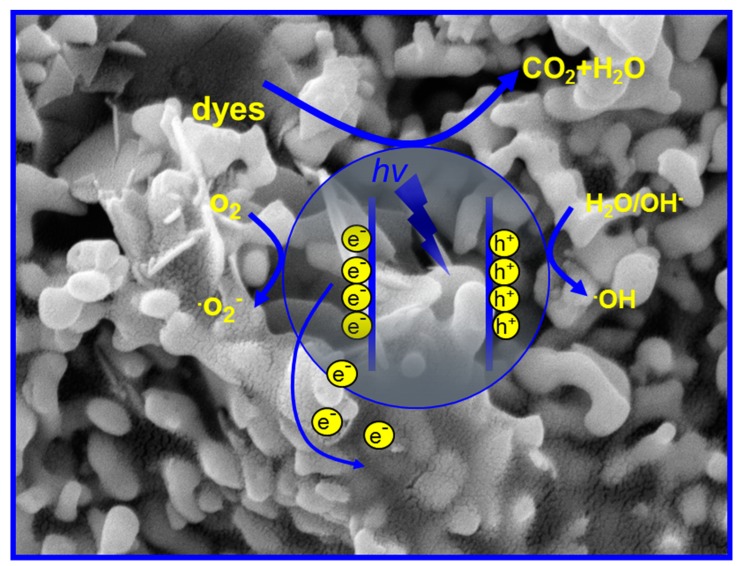
Speculation schematic of β-Bi_2_O_3_/GN photocatalytic degradation mechanism of dye.

According to the above experimental results, the possible photocatalytic mechanisms of β-Bi_2_O_3_/GN were estimated, as shown in Figure 6. Under simulated sunlight irradiation, the excited electrons of the β-Bi_2_O_3_ semiconductor divert a part of the electrons out due to the good conductivity of the graphene layers in the course of being conducted to the conduction band. In other words, in the system of β-Bi_2_O_3_/GN, the graphene, as the receiving body and conductor of the electrons, effectively separated the photogenerated electron-hole pairs to avoid the recombination of electrons and holes. It’s similarly compared with a previously studied system in which the GO embedded into TiO_2_ nanofiber and served as a conduit of electron transfer [25]. Posa et al. [10] and Li et al. [23] prepared graphene–TiO_2_ nanocomposite and CdS-cluster-decorated graphene nanosheets respectively; the similar mechanisms were proposed, too. Furthermore, the unique features of graphene also contribute to the improvement of photocatalytic activity, which allow photocatalytic reactions to take place not only on the surface of semiconductor catalysts, but also on the graphene sheet, greatly enlarging the reaction space.

## 4. Conclusions

With the simple method of solution mixing, β-Bi_2_O_3_/GN composite photocatalysts with different qualities were successfully prepared. In the test of degradation rate, it was found that the sample with 1% GN on its surface had the highest photocatalytic activity, with its dye degradation efficiency being three times higher than the pure β-Bi_2_O_3_. However, when the content of GN increased, the degradation rate decreased. This was because the introduction of more graphene may decrease the light absorption of the reaction solution. Overall, the introduction of appropriate quantity of graphene could significantly increase the photocatalytic degradation rate of the catalyst.

## Supplementary Materials

The following are available online at http://www.mdpi.com/1996-1944/11/8/1359/s1, Figure S1: The emission spectrum of a 300-W Xenon lamp (CEL-HXF300, AULTT, Beijing, China).
