# Supplementary material for: Facile Fabrication of Dumbbell-Like β-Bi2O3/Graphene Nanocomposites and Their Highly Efficient Photocatalytic Activity
Source: Materials (Basel). 2018 Aug 6;11(8):1359. doi: 10.3390/ma11081359 (PMC6119887; doi:10.3390/ma11081359)
Supplement: Supplementary file 1 [file materials-11-01359-s001.zip › materials-333907-SI.pdf]

Supplementary materials

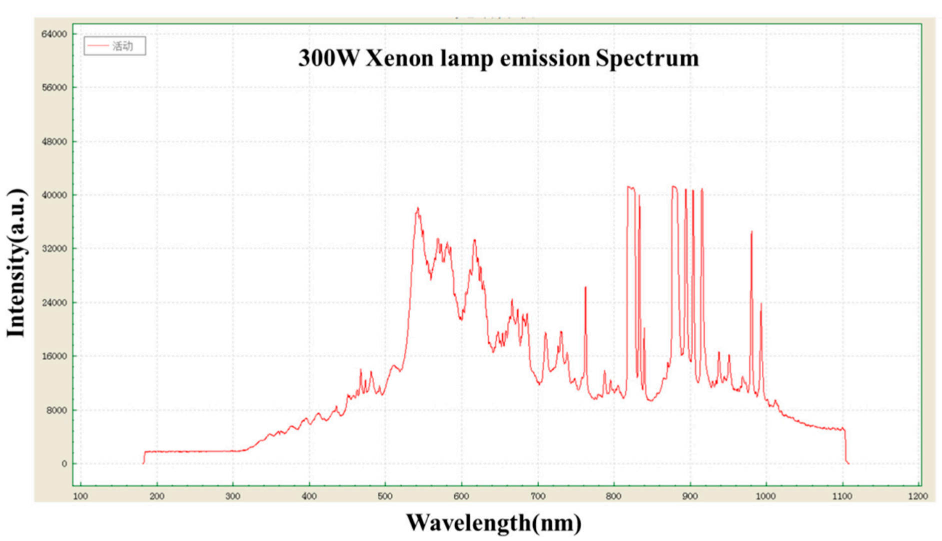

Figure S1. The emission spectrum of a 300-W Xenon lamp (CEL-HXF300, AULTT, Beijing, China).
